# Trust in the healthcare system and mortality: A population-based prospective cohort study in southern Sweden

**DOI:** 10.1016/j.ssmph.2022.101109

**Published:** 2022-04-27

**Authors:** Martin Lindström, Mirnabi Pirouzifard

**Affiliations:** Social Medicine and Health Policy, Department of Clinical Sciences and Centre for Primary Health Care Research, Lund University, S-205 02, Malmö, Sweden

**Keywords:** Trust in the healthcare system, Generalized trust in other people, Social capital, Health-related behaviors, Mortality, Cardiovascular mortality, Cancer mortality, Sweden

## Abstract

**Aims:**

To investigate associations between trust in the healthcare system and all-cause, cardiovascular, cancer and other causes mortality.

**Study design:**

Prospective cohort study.

**Methods:**

A public health questionnaire was conducted in 2008 in Scania, the southernmost part of Sweden, with a 54.1% participation rate with a postal questionnaire and three reminders. In this study 24,833 respondents were included. The baseline questionnaire study was linked to prospective 8.3-year follow-up cause-specific mortality register data. Survival (Cox) regression analyses were conducted.

**Results:**

A 15.2% proportion of respondents reported very high, 59.1% rather high, and 21.7% not particularly high trust in the healthcare system, while 3.2% reported no trust at all and 0.9% did not know. The groups with rather high and not particularly high trust in the healthcare system had significantly lower all-cause mortality than the reference group with very high trust in the healthcare system. These statistically significant results remained throughout the multiple analyses, and were explained by lower cancer mortality in both the rather high and not particularly high trust respondent groups, and lower cardiovascular mortality in the not particularly high trust respondent group. No significant results were observed in the adjusted models for other causes mortality. No significant results were observed for the no trust and don't know categories in the multiple adjusted models, but these groups are small.

**Conclusions:**

The results suggest a comparative advantage of moderate trust compared to very high trust in this setting of long waiting times for cancer and CVD treatment.

## Highlights

• Trust in the healthcare system and mortality was investigated.
• Rather high and not particularly high trust groups had lower all-cause mortality than the very high trust group.
• The lower mortality was mainly explained by lower cancer mortality.
• The lower mortality was partly explained by lower CVD mortality.
• The findings are associated with long-term queueing for particularly cancer treatment.

## 1 Introduction

Trust in healthcare and the healthcare system is regarded as a valid instrument for the evaluation of healthcare performance (Chang et al., 2013; Straten et al., 2002). A population-based study from the county of Stockholm in 2008 regarding reasons to file complaints and actually having filed such a complaint suggested that the number of formal complaints made regarding the healthcare system was increasing although still small compared to the number of grievances. The authors concluded that the number of actually officially filed complaints were just the top of an iceberg (Wessel et al., 2012). Self-reported trust in healthcare providers and the healthcare system may thus give a more valid overall view of healthcare performance than the rate of formal complaints.

Trust in contact with healthcare and the healthcare system is commonly defined as the acceptance of a vulnerable situation where the patient or care receiver has confidence that the healthcare provider will care for the patient's/care receiver's best interests (Hall et al., 2001). Trust in the healthcare provider may have many positive effects. Trust may modify patient attitude and behavior, improve the placebo effect, enhance acceptance of medical suggestions, improve compliance with treatment recommendations, decrease the risk of low compliance with medication due to cost pressure, increase motivation to seek help, and increase motivation for preventive measures. Trust in the healthcare provider may also enhance communication between caregivers and patients, and improve perceptions of efficacy, self-reported health status, well-being and quality of life (Lewandowski et al., 2021). Still, trust in healthcare and the healthcare system is based on an asymmetry between caregiver and patient based on the caregiver's profession (Arrow, 1963; Freidson, 2001). An information gradient is obvious, and a potential power gradient is possible to readily discern.

Low trust in healthcare is not only an issue of asymmetry in information and power between an individual patient/user and an individual healthcare provider. Trust may be regarded both as an interpersonal and as a social cognitive characteristic. It may pertain to individuals as well as organizations and public institutions (LoCurto & Berg, 2016; Mechanic, 1996). Topp and Chipukuma (2016) qualitatively described a chain of low trust originating at the top of the healthcare system starting with inadequate resources and suboptimal leadership leading to low trust in healthcare providers in their workplace, low quality and/or delayed service, and eventually low level of trust between provider and patient (Topp & Chipukuma, 2016). In the literature on social capital and health, trust across such an asymmetrical information and power gradient is referred to as institutional, vertical or linking trust, linking individual citizens/inhabitants to the public institutions of society. Social capital and trust has both a horizontal dimension, i.e. generalized trust in other people in society without an information/power gradient, and a vertical dimension, i.e. individuals' trust in public institutions across an information and power gradient (Narayan, 2002; Narayan & Cassidy, 2001). Social capital is defined as features of social structures, such as interpersonal trust, norms of reciprocity and mutual aid, which constitute resources to facilitate interaction between individuals and groups in order to enhance collective action (Coleman, 1990; Putnam, 1993). Institutional trust or vertical trust concerns especially citizens' trust in the public institutions within society (Veenstra & Lomas, 1999). Earlier studies have e.g. shown cross-sectional associations between high institutional trust in the *Riksdag* (the Swedish parliament) and high self-rated health (Mohseni & Lindström, 2008) and high psychological health (measured with GHQ12) (Lindström & Mohseni, 2009).

Several studies indicate a positive association between trust in healthcare providers and self-assessed health-related quality of life (HRQoL) (AlRuthia et al., 2020; Muirhead et al., 2014), medication adherence (Linetzky et al., 2017; Zolnierek & Dimatteo, 2009), ability of self-managament among persons with chronic conditions (Cramm & Nieboer, 2015), breast cancer screening (Hong et al., 2018), and rates of colorectal cancer screening (Carcaise-Edinboro & Bradley, 2008). In contrast, studies regarding trust in healthcare and objective health outcomes have shown no or weaker associations. The association between trust in healthcare and health outcomes was in a meta-analysis concluded to be small to moderate. The results suggested moderate associations between trust in healthcare and self-rated subjective outcomes but no significant associations between trust in healthcare and objective health outcomes, e.g. blood pressure, HbA1c, BMI, and observer-rated effects, e.g. diagnosis by a professional (Birkhauer et al., 2017). Crowdsourced health care facility ratings were associated with mortality in US counties in a study with ecological study design (Stokes et al., 2021), but no study has to our knowledge prospectively analyzed associations between self-reported trust in healthcare and mortality based on individual level data. This study will analyze associations between self-reported trust in the healthcare system and mortality in 2008 in Scania in the southernmost part of Sweden. The 2008 public health survey will be linked to prospective all-cause, cardiovascular (CVD), cancer and other causes mortality in a population-based 8.3-year prospective cohort study among 18–80 year old respondents. Following previous studies, the hypothesis is that high trust in the healthcare system is associated with lower all-cause, CVD, cancer and other causes mortality, adjusting for relevant factors including demographics, socioeconomic status (SES), chronic disease at baseline, health-related behaviors and generalized (horizontal) trust in other people. The null hypothesis is that there are no such associations. Earlier studies based on the cross-sectional public health questionnaire in 2004 yielded significantly lower odds ratios of both tobacco smoking, and higher odds ratios of having quit smoking if ever smoker for respondents with high trust in the healthcare system (Lindström & Janzon, 2007), and a significant association between low trust in the healthcare system and poor self-rated health (Mohseni & Lindström, 2007).

The aim of this study is to longitudinally investigate associations between trust in the healthcare system and all-cause, CVD, cancer and other causes mortality in a prospective cohort study, adjusting for relevant demographics, SES, baseline chronic disease, health-related behaviors and generalized trust in other people.

## 2 Material and methods

### 2.1 Study population

The public health survey in Scania 2008 is a cross-sectional survey based on a stratified sample of the official register population aged 18–80 which was conducted in Scania (Skåne), the southernmost part of Sweden, in the autumn of 2008. A postal invitation letter, which included a questionnaire, was sent, followed by three postal reminders to initial non-respondents. The questionnaire was also possible to complete online. Exactly 28,198 responded, yielding a 54.1% participation rate. The baseline questionnaire study was performed by Region Skåne which is the regional authority responsible for the healthcare system in Scania. The questionnaire includes 134 items concerning demographic and social conditions, self-reported health, self-reported psychological health, psychosocial conditions, social support, social capital, physical and psychosocial work conditions, health-related behaviors and crime and security related items. The random sample was stratified into 29 smaller municipalities and city parts in the four major cities, age, sex and education in order to get statistical power in all 60 geographical areas (municipalities and city parts). Statistical power is thus sufficient in the present study of trust in the healthcare system and mortality. The stratified sample was created by Statistics Sweden. Statistics Sweden also created the population weight compensating for the stratification of the sample in all analyses in this study. The population weight accounts for lower response rates among men, younger people, people born abroad and people with less education. The cross-sectional baseline questionnaire data from 2008 was linked to data concerning prospective mortality obtained from the National Board of Health and Welfare (*Socialstyrelsen*).

The present study links the baseline questionnaire data from 2008 to prospective mortality data from official population death register. It was approved by the Ethical Committee (*Etikprövningsnämnden*) in Lund (No. 2010/343).

### 2.2 Dependent variables

Mortality was followed prospectively from 27 August-14 November 2008 (according to registration date of individual answers) until 31 December 2016 (8.3 years onwards), or until death. A total of 24,833 participants were included in this study, 11,307 men and 13,526 women, excluding 3093 respondents with internally missing values on any or several of the variables analyzed in this study, while 136 respondents were lost to follow-up. The causes of death were coded according to ICD10. The Swedish ten-digit person number system makes linkage of baseline data from the 2008 survey with the Swedish national causes of death register at the Swedish *National Board on Health and Welfare* by a third party (private company) possible. The ten-digit person numbers were deleted before delivery from the *National Board of Health and Welfare* to the researchers.

All-cause (total), cardiovascular (I00-I98), cancer (C00-C97), and all other causes (other causes than I00-I98 and C00-C97) mortality were analyzed. All-cause mortality is the total sum of the other three cause-specific mortality categories.

### 2.3 Independent variables

*Trust in the healthcare system* was assessed with the item “What trust do you have in the healthcare system?” with the alternative answers “Very high trust”, “Rather high trust”, “Not particularly high trust”, “No trust at all” and “Do not know”.

**Fig. 1:**
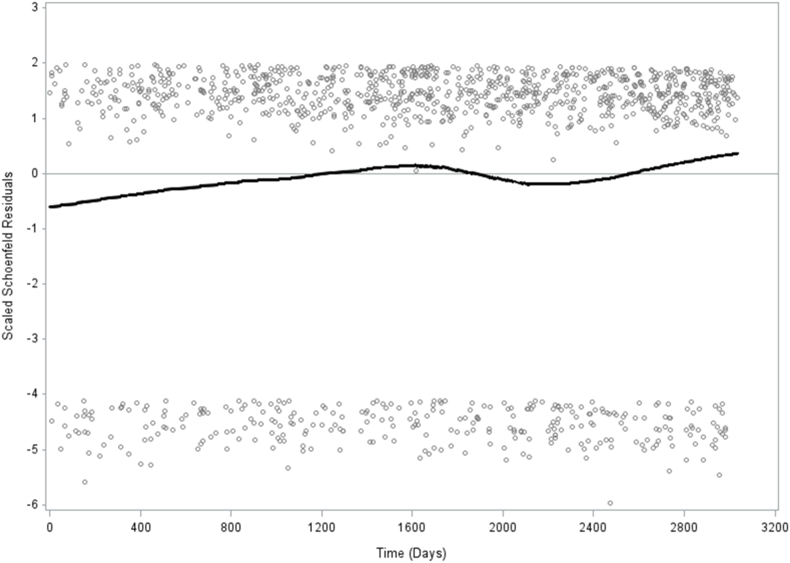
We performed the proportionality test after removing individuals with no trust and don't know alternatives (866 individuals) from the variabletrust in the healthcare system. A total 23967 persons remained for the proportionality test. Then we created a new variable with two groups by placing individuals with Very high trust in one group, and those with rather high trust and not particularly high trust in the healthcare system in the second group. With this categorization, there are 3788 respondents (15.8%) with very high trust and 20179 respondents (84.2%) with rather high/not particularly high trust in the healthcare system. The total number of deaths decreased from 1265 to 1227 individuals by just using these three categories. The P-value for proportionality based on interaction term between trust in the healthcare system and time of follow-up is 0.436.

Men and women were collapsed in all analyses in Table 1, Table 2, Table 3 and Fig. 1. Analyses in Table 2, Table 3 were adjusted for sex.

**Table 1:** Descriptive characteristics (%) of age, sex, socioeconomic status (SES), country of birth, chronic disease, low leisure-time physical activity, smoking, alcohol consumption, and generalized trust in other people by trust in the healthcare system.

	Trust in the healthcare system (F84A)					p-value
	Very high	Rather high	Not high	No trust	Don't know	
	n = 3788	n = 15107	n = 5072	n = 671	n = 195	
	15.2%	59.1%	21.7%	3.2%	0.9%	
**Age**, yrs: mean ± SD a	46.6 ± 18.3 (45.8–47.3)	46.5 ± 16.4 (46.2–46.8)	44.6 ± 16.3 (44.0–45.1)	40.9 ± 16.4 (39.5–42.2)	44.6 ± 19.2 (41.2–48.0)	<0.001
**Sex**^b^						
Male	58.8 (56.8–60.7)	48.8 (47.8–49.9)	46.8 (44.9–48.6)	57.0 (52.2–61.7)	49.8 (40.7–59.0)	
Female	41.2 (39.3–43.2)	51.2 (50.1–52.2)	53.2 (51.4–55.1)	43.0 (38.3–47.8)	50.2 (41.0–59.3)	
**Socioeconomic status (SES)**^b^						<0.001
Higher non-manual	8.1 (7.1–9.0)	9.9 (9.3–10.5)	7.9 (7.1–8.8)	6.9 (4.9–8.9)	2.6 (0.2–5.0)	
Medium non-ma	11.9 (10.8–13.1)	15.5 (14.8–16.2)	12.3 (11.2–13.4)	8.5 (6.1–10.9)	7.5 (3.4–11.6)	
Lower non-manual	6.4 (5.4–7.3)	8.3 (7.8–8.9)	8.4 (7.5–9.3)	8.2 (5.5–10.9)	4.2 (1.0–7.4)	
Skilled manual	7.5 (6.5–8.6)	11.0 (10.3–11.6)	11.6 (10.5–12.7)	10.5 (7.4–13.5)	7.3 (2.5–12.2)	
Unskilled manual	12.6 (11.2–13.9)	12.3 (11.6–13.0)	13.5 (12.3–14.7)	12.6 (9.5–15.7)	16.3 (9.9–22.7)	
Self-employed/farmer	5.5 (4.6–6.4)	5.5 (5.1–6.0)	7.3 (6.4–8.2)	10.7 (7.8–13.5)	2.6 (0.0–5.2)	
Early retired	4.4 (3.5–5.3)	3.3 (2.9–3.7)	4.4 (3.7–5.0)	5.4 (3.2–7.5)	4.3 (1.5–7.2)	
Unemployed	3.9 (3.0–4.8)	3.3 (2.9–3.7)	5.3 (4.4–6.1)	6.1 (3.5–8.7)	8.1 (3.4–12.8)	
Student	11.2 (9.7–12.7)	7.3 (6.7–7.9)	8.4 (7.3–9.5)	10.5 (7.0–14.1)	15.9 (7.4–24.3)	
Old age pensioner	22.1 (20.7–23.6)	17.8 (17.1–18.4)	13.0 (12.0–14.0)	9.4 (7.0–11.8)	21.8 (14.5–29.2)	
Unclassified	5.7 (4.7–6.8)	4.7 (4.3–5.2)	6.4 (5.5–7.4)	9.4 (6.0–12.7)	7.9 (3.0–12.9)	
Long-term sickleave	0.7 (0.4–1.0)	1.1 (0.9–1.3)	1.4 (1.0–1.9)	1.9 (0.7–3.1)	1.5 (0.0–3.7)	
**Country of birth**^b^	16.9 (15.1–18.6)	14.0 (13.2–14.8)	24.9 (23.3–26.5)	33.5 (28.2–38.8)	43.8 (34.3–53.3)	<0.001
**Chronic disease**^b^	28.7 (26.9–30.6)	26.7 (25.8–27.5)	31.4 (29.9–33.0)	41.5 (36.8–46.3)	24.1 (17.0–31.1)	<0.001
**Low leisure-time physical activity**^b^	12.8 (11.6–14.1)	12.0 (11.4–12.7)	17.2 (15.8–18.6)	27.3 (22.7–31.9)	30.8 (22.2–39.4)	<0.001
**Smoking**^b^	10.5 (9.2–11.7)	13.3 (12.6–14.0)	18.0 (16.6–19.4)	23.0 (18.7–27.1)	27.6 (19.1–36.1)	<0.001
**Alcohol drinking past year**^b^						<0.001
Never	14.4 (12.9–15.9)	9.3 (8.7–9.9)	13.0 (11.8–14.2)	19.4 (15.2–23.7)	32.0 (22.7–41.3)	
Once a month or more seldom	22.7 (21.0–24.4)	21.8 (21.0–22.7)	24.6 (23.1–26.1)	26.3 (22.1–30.5)	29.0 (20.6–37.4)	
2-4 times a month	35.3 (33.4–37.2)	37.2 (36.3–38.2)	34.4 (32.8–36.0)	29.5 (24.9–34.1)	24.7 (17.3–32.2)	
2-3 times a week	20.8 (19.3–22.3)	24.2 (23.3–25.0)	20.9 (19.6–22.2)	17.2 (13.6–20.8)	9.8 (4.9–14.7)	
At least 4 times a week	6.8 (5.9–7.7)	7.5 (7.0–7.9)	7.1 (6.3–7.9)	7.6 (5.3–9.8)	4.5 (1.4–7.7)	
**Low generalized trust in other people**^b^						<0.001
	28.0 (26.2–29.9)	32.6 (31.6–33.6)	48.9 (47.2–50.6)	61.9 (57.2–66.6)	61.1 (52.6–69.6)	

The 2008–2016 Public Health Survey of Scania, Sweden. Total population n = 24833. Weighted prevalence.

The values in parentheses are 95% confidence intervals for mean or percent based on bootstrap method with 1000 number of replicates.

a p-value: Independent samples ANOVA-test, 2-tailed.

b p-value: Pearson Chi Square test, 2-sided.

**Table 2:** Hazard rate ratios (HRRs) from Cox regression models for all-cause mortality and cause-specific mortality, showing associations with trust in the healthcare system.

	Model 0		Model 1		Model 2		Model 3		Model 4		
Cause of death	HRR	(95%CI)	HRR	(95%CI)	HRR	(95%CI)	HRR	(95%CI)	HRR	(95% CI)	Number of Deaths
**All causes**											1265
Very high trust	1.0		1.0		1.0		1.0		1.0		
Rather high trust	0.6***	(0.5–0.7)	0.8**	(0.7–0.9)	0.8*	(0.7–1.0)	0.8*	(0.7–1.0)	0.8*	(0.7–1.0)	
Not particularly high	0.4***	(0.3–0.5)	0.7***	(0-5-0.8)	0.6***	(0.5–0.8)	0.6***	(0.5–0.8)	0.6***	(0.5–0.7)	
No trust	0.5**	(0.3–0.8)	1.2	(0.7–1.9)	1.0	(0.6–1.7)	0.8	(0.5–1.3)	0.8	(0.5–1.2)	
Do not know	0.5	(0.2–1.3)	0.6	(0.2–1.6)	0.5	(0.2–1.6)	0.4	(0.1–1.2)	0.4	(0.1–1.2)	
**Cardiovascular disease**											372
Very high trust	1.0		1.0		1.0		1.0		1.0		
Rather high trust	0.6**	(0.5–0.9)	0.9	(0.6–1.2)	0.9	(0.7–1.2)	0.9	(0.7–1.3)	0.9	(0.7–1.3)	
Not particularly high	0.4***	(0.3–0.6)	0.7	(0.4–1.0)	0.6*	(0.4–1.0)	0.6*	(0.4–0.9)	0.6*	(0.3–0.9)	
No trust	0.2	(0.0–6.9)	0.5	(0.0–18.6)	0.4	(0.0–16.7)	0.3	(0.0–13.2)	0.3	(0.0–13.0)	
Do not know	0.9	(0.1–12.1)	1.2	(0.1–17.1)	1.1	(0.1–17.0)	0.8	(0.1–12.0)	0.8	(0.1–11.9)	
**Cancer**											499
Very high trust	1.0		1.0		1.0		1.0		1.0		
Rather high trust	0.6***	(0.5–0.8)	0.7**	(0.5–0.9)	0.7**	(0.5–0.9)	0.7**	(0.5–0.9)	0.7**	(0.5–0.9)	
Not particularly high	0.4***	(0.2–0.5)	0.5**	(0.4–0.8)	0.5**	(0.3–0.8)	0.5***	(0.3–0.7)	0.5***	(0.3–0.7)	
No trust	0.3*	(0.1–0.9)	0.7	(0.3–1.9)	0.7	(0.3–1.8)	0.6	(0.2–1.6)	0.5	(0.2–1.4)	
Do not know	0.4	(0.0–4384.9)	0.5	(0.0–4934.3)	0.5	(0.0–5006.0)	0.4	(0.0–4452.1)	0.4	(0.0–4204.7)	
**Others**											394
Very high trust	1.0		1.0		1.0		1.0		1.0		
Rather high trust	0.7*	(0.5–0.9)	0.8	(0.6–1.1)	0.9	(0.7–1.2)	0.9	(0.7–1.2)	0.9	(0.7–1.2)	
Not particularly high	0.6**	(0.4–0.9)	0.9	(0.6–1.3)	0.8	(0.5–1.2)	0.8	(0.5–1.2)	0.7	(0.5–1.1)	
No trust	1.1	(0.5–2.1)	2.5*	(1.2–4.9)	2.0	(1.0–4.2)	1.5	(0.7–3.0)	1.4	(0.7–2.9)	
Do not know	0.1	(0.0–11.7)	0.1	(0.0–16.6)	0.1	(0.0–15.3)	0.1	(0.0–9.9)	0.1	(0.0–9.7)	

The 2008–2016 Scania public health survey with 8.3 years follow-up.

Men and women combined. Total population n = 24833. **Weighted.**

Model 0 unadjusted.

Model 1 adjusted for sex and age.

Model 2 furthermore adjusted for socioeconomic status, country of birth and chronic disease.

Model 3 furthermore adjusted for leisure-time physical activity, smoking and alcohol consumption.

Model 4 furthermore adjusted for generalized trust in other people.

Significance levels: *p < 0.05, **p < 0.01, ***p < 0.001.

Weighted Hazard Ratios. Bootstrap method (1000 replicates) for variation estimation.

**Table 3:** Hazard rate ratios (HRRs) from Cox regression models for all-cause mortality, showing associations with trust in the healthcare system.

	Model 4							
	1.3-year follow-up (n = 127^£^)		2.3-years follow-up(n = 242^£^)		3.3-years follow-up(n = 389^£^)		4.3-years follow-up(n = 537^£^)	
Cause of death	HRR	(95% CI)	HRR	(95% CI)	HRR	(95% CI)	HRR	(95% CI)
**All causes**								
Very high trust = REF	1.0		1.0		1.0		1.0	
Rather high trust	0.6	(0.4–1.1)	0.7	(0.5–1.1)	0.7*	(0.5–1.0)	0.8	(0.6–1.1)
Not particularly high	0.5	(0.2–1.1)	0.6	(0.3–1.1)	0.6*	(0.4–1.0)	0.6**	(0.4–0.9)
No trust	0.9	(0.1–7.8)	1.2	(0.5–2.7)	1.1	(0.5–2.1)	0.9	(0.5–1.8)
Do not know	NA	NA	0.4	(0.0–3*10^3^)	0.9	(0.1–6.1)	0.7	(0.1–4.9)

The 2008–2016 Scania public health survey with 1.3, 2.3, 3.3- and 4.3-years follow-up.

Men and women combined. Total population n = 24833. **Weighted prevalence.**

Model 4 Adjusted for sex, age, socioeconomic status, country of birth, chronic disease, leisure-time physical activity, smoking, alcohol consumption and generalized trust in other people.

Significance levels: *p < 0.05, **p < 0.01, ***p < 0.001.

Weighted Hazard Ratios. Bootstrap method (1000 replicates) for variation estimation.

^£^Number of deaths.

Abbreviation: NA, Not Available; HR, Hazard Ratio.

*Age* was included as a continuous variable.

*Country of birth* was categorized as born in Sweden or in other country.

*Socioeconomic status (SES)* (by occupation and relation to labor market) was divided into non-manual employees in higher, medium and lower positions, skilled and unskilled manual workers, and self-employed/farmers. The categories outside the workforce entail the unemployed (job seekers), students, early retired (before age 65), long-term sick leave, pensioners aged 65-, and unclassified.

*Chronic disease* was assessed with the item “Do you have any long-term disease, ailment or injury, any disability or other weakness?”, and entailed the alternative answers “Yes” and “No”.

*Leisure-time physical activity* (LTPA) was obtained with the four alternatives regular exercise (at least three times per week at least 30 min/occasion, leading to sweating), moderate regular exercise (exercising once or twice per week at least 30 min/occasion, leading to sweating), moderate exercise (walking, cycling or equivalent activity status in leisure-time less than 2 h walking, cycling or equivalent activity/week) and sedentary status (less than 2 h walking, cycling or equivalent activity/week). The first three alternatives were collapsed as high LTPA and the fourth was defined as low.

*Tobacco smoking* was obtained with the item “Do you smoke?” with the alternatives daily, non-daily and non-smoker, collapsing the first two alternatives, which yielded “smoking” and “non-smoking”.

*Alcohol consumption* was assessed with the item “How often have you consumed alcohol during the past twelve months?” with the options “4 times per week or more”, “2–3 times per week”, “2–4 times per month”, “Once per month or more seldom”, and “Never”.

*Generalized trust in other people* was assessed with the item “Most people can be trusted”, with the alternative answers “Do not agree at all”, “Do not agree”, “Agree” and “Agree completely”. This item was dichotomized with the two first alternatives collapsed as “low trust” and the two latter alternatives collapsed as “high trust”.

### 2.4 Statistics

Prevalence (%) of all variables were analyzed based on the five categories of the trust in healthcare system item. Differences between the five trust in the healthcare system categories were analyzed using ANOVA test for continuous variables and chi-square test for categorical variables (p-values) (Table 1). Hazard rate ratios (HRR:s) with 95% confidence intervals (95% CI:s) of all-cause, cardiovascular, cancer and other causes mortality by trust in the healthcare system were calculated. Five models were calculated: model 0 unadjusted, model 1 adjusted for sex and age, model 2 adjusted for sex, age, country of birth, SES, and chronic disease, model 3 additionally adjusted for LTPA, tobacco smoking, alcohol consumption, and model 4 additionally adjusted for generalized trust in other people (Table 2). Sensitivity analyses were conducted with 1.3-year, 2.3-year, 3.3-year and 4.3-year follow-up time mortality, adjusted for all covariates included in model 4 above, in order to assess whether effect measures remain similar over time in terms of strength and direction of association (Table 3). Follow-up days were assessed from baseline to death or last follow-up date (2016-12-31), depending on which occurred first. Bootstrap analysis makes investigation of the sampling variability without distributional assumptions of the study population possible (SAS/STAT Software Survey Analysis, 2021). To ensure accurate variance estimation on weighted data, bootstrap methods with 1000 numbers of replicates to obtain confidence intervals and p-values were used. Test of proportionality for trust in the healthcare system and mortality was conducted. The assumption of proportional hazards was determined by introducing an interaction term with time and trust in the healthcare system. Schoenfeld residuals were analyzed for trust in the healthcare system and mortality, comparing the respondent group with very high trust to the two collapsed respondent groups with rather high and not particularly high trust in the healthcare system (Fig. 1). To perform calculations, the SAS software version 9.4 was utilized.

## 3 Results

Table 1 shows that 15.2% of respondents reported very high, 59.1% rather high, and 21.7% not particularly high trust in the healthcare system, while 3.2% reported no trust at all and 0.9% did not know. Mean age was significantly lower in the not particularly high and no trust categories compared to the very high trust category. Men were overrepresented in the very high trust and no trust categories. SES differences between the five trust in the healthcare system categories were comparatively small, but the rather high trust category had significantly higher proportions of high non-manual and medium level non-manual employees than the other categories. The proportion of persons born abroad was lowest in the rather high trust category and increased significantly and strongly in the not particularly high trust, no trust and do not know categories. A significantly higher proportion of respondents in the not particularly high trust and no trust categories reported chronic disease compared to the very high trust and rather high trust categories. The proportions with low LTPA increased significantly with decreasing trust in the healthcare system in the not particularly high trust, no trust and do not know categories compared to the very high trust and rather high trust categories. The proportions with smoking increased significantly with decreasing trust and the do not know category compared to the very high trust in the healthcare system category. Alcohol consumption during the past year also displayed significant differences between the five categories of trust in the healthcare system, e.g. a high proportion of never drinkers during the past year in the do not know category. Low generalized trust in other people increased significantly and continually from 28,0% (26.2%–29.9%) in the very high trust in the healthcare system category, 32.6% (31.6%–33.6%) in the rather high trust category, 48.9% (47.2%–50.6%) in the not particularly high trust category, 61.9% (57.2%–66.6%) in the no trust and 61.1% (52.6%–69.6%) in the do not know category.

Table 2 shows that all-cause mortality remained significantly lower for the rather high trust (2) and not particularly high trust (3) categories compared to the very high trust in the healthcare system category (1) throughout the analyses (models 0 to 4). In the final fully adjusted model 4 the rather high trust category displayed an HRR 0.8 (0.7–1.0) and the not particularly high trust an HRR 0.6 (0.5–0.7) of all-cause mortality compared to the very high trust in the healthcare system category. The not particularly high trust in the healthcare system category displayed lower HRRs for CVD, HRR 0.6 (0.3–0.9), and cancer, HRR 0.5 (0.3–0.7), in the final model 4 compared to the very high trust in the healthcare system category. The rather high trust in the healthcare system category displayed significantly lower cancer mortality throughout the analyses, with HRR 0.7 (0.5–0.9) in the final model 4 compared to the very high trust in the healthcare system category. No significant differences for other causes of death were observed in models 2–4.

Table 3 shows that the HRRs remained similar in strength and direction for the 1.3-year, 2.3-year, 3.3-year and 4.3-year follow-up periods compared to the HRRs for the 8.3-year follow-up when analyzing model 4 with all covariates for these four shorter follow-up periods regarding all-cause mortality. The HRRs became statistically significant for rather high trust, HRR 0.7 (0.5–1.0), and not particularly high trust, HRR 0.6 (0.4–1.0), in model 4 for the 3.3-year follow-up, and for not particularly high trust, HRR 0.6 (0.4-0-9), for the 4.3-year follow-up.

Fig. 1 shows that the Schoenfeld residuals for trust in the healthcare system and all-cause mortality were stable and consistent over time when the respondent group with very high trust was compared to the two collapsed respondent groups with rather high and not particularly high trust in the healthcare system. The interaction term between trust in the healthcare system and mortality across the 8.3 year period was not significant, p = 0.436.

## 4 Discussion

The results of this study were initially not even hypothesized, i.e. neither the hypothesis nor the null hypothesis were confirmed, since the groups with rather high and not particularly high trust in the healthcare system had significantly lower (instead of higher) all-cause mortality than the reference group with very high trust in the healthcare system. These statistically significant results remained essentially unaltered throughout the multiple analyses including the final full model with generalized (horizontal) trust in other people included. These stable results were explained by significantly lower cancer mortality in the respondent groups with rather high and not particularly high trust in the healthcare system, and also significantly lower CVD mortality in the respondent group with not particularly high trust in the healthcare system. No statistically significant results were observed in the three most fully adjusted models for other causes mortality. No significant results were observed for the no trust and don't know categories in the multiple adjusted models, but numbers for these groups were small and thus hard to interpret due to lack of statistical power. The results may not be fully generalizable to settings with no shortage of specific aspects of healthcare such as e.g. comparatively long-term queueing for cancer treatment.

The results suggest a comparative advantage of moderate trust compared to very high trust in a setting characterized by restricted access to treatment particularly with regard to cancer treatment. Neither the original hypothesis nor the null hypothesis were fulfilled. The results may seem paradoxical but may be understandable in the political and administrative context of Region Scania, responsible for the healthcare system in southernmost Sweden. For more than ten years the Swedish healthcare system in general as well as Region Scania has had problems with long queueing, particularly for treatment of patients with several important cancer diagnoses. The most common cancer diagnoses involved in this logistic queueing problem are prostate cancer and breast cancer, which also indicates that men and women are affected by long waiting times to a similar extent. Similar logistic problems with queueing beyond the guaranteed maximal waiting time have also existed for a long time regarding elective treatment of CVD (Ghazvinian et al., 2021), although this has been less debated. For this reason, cancer patients in Scania in particular have been treated abroad (Dagens Medicin, 2019) and private alternatives have appeared for groups with relevant insurance or private means to circumvent parts of the queueing (Trysell, 2020). Extensive privatizations of healthcare have also been conducted in the primary healthcare system (Lindström et al., 2018).

Actors place their trust in others (horizontal trust) based on both judgement and performance of equal others after rational considerations. This is also applicable to institutional (vertical) trust across an information and power gradient (Coleman, 1990). Political trust, i.e. trust in politicians, is important for democracy. People should trust each other and the government, and be able to come together in order to solve problems within a framework of constructive political debate followed by cooperation (Uslaner, 2002). However, democracy also presumes some light to moderate degree of distrust and questioning of authorities across an information and power gradient as well. Consent in a democratic order is based on the fact that political and administrative leaders do keep their promises, perform competently and treat their citizens well (Levi, 1998; Warren, 1996). The same mechanisms may be at work in our main results yielding significantly lower cancer mortality among respondents with rather high and not particularly high trust in the healthcare system compared to the very high trust reference group. Very high trust in the healthcare system seems to be most rational if healthcare providers and the healthcare system are performing outstandingly well over long time. In a high tax system such as Sweden with important aspects of performance below levels which could be reasonably expected, rather high and not particularly high trust may be more rational and increase the incentive to find alternative solutions to comparatively long-term queueing within the tax-financed healthcare system. Lowered levels of trust in the healthcare system would also rationally increase the propensity to question information and judgements made within the healthcare system. Patients active in this regard may thus gain an advantage also within the healthcare system compared to patients with very high trust in the healthcare system who would be more likely to merely accept information and waiting times without questioning, and less likely to seek alternatives. The mechanisms may be similar with regard to CVD mortality, although the patterns are less pronounced and not statistically significant for the category rather high trust in the healthcare system.

The findings that baseline respondents with rather high and not particularly high trust in the healthcare system have significantly lower all-cause mortality mainly caused by significantly lower cancer mortality (and partly CVD mortality) are plausibly not generalizable to all countries and all populations. In particular, they do not apply to countries with healthcare systems that are performing outstandingly or at least expectedly well. Under such circumstances very high trust in the healthcare system may be fully rational and a mindset to be expected. Still, the results may plausibly be generalizable to healthcare systems that work sub-optimally or are literally underperforming in relation to what could reasonably be expected. However, this assumption of what to expect can only be verified by further preferably longitudinal studies from Sweden and other countries.

The basis for social capital and health research is the assumption that social capital promotes health through several causal mechanisms (Kawachi et al., 1999). However, it is also apparent that both vertical and horizontal social capital may have a dark side with adverse effects on health including e.g. social exclusion, discrimination, bullying and social ostracism (Villalonga-Olives & Kawachi, 2017). However, a condition in which social capital and trust are optimal for the individual if adjusted by moderation in response to external conditions is also apparent (Uslaner, 2002). The moderation of institutional (or vertical) trust may also possibly be extended to generalized trust in other people (or horizontal trust). How suitable is it for instance to be among the few per cent with high generalized (horizontal) trust in others in countries with a very low prevalence (%) of high trust in others between citizens (Inglehart et al., 1998)?

Ecological studies displaying associations between health care facility ratings and mortality (Stokes et al., 2021) may include the risk of drawing causal conclusions based on the ecological fallacy, i.e. conclusions regarding individual characteristics and associations based exclusively and erroneously on geographic area level data **(**Diex-Roux, 1998; Schwartz, 1994**).** The present study, in contrast, investigates the associations between trust in the healthcare system and mortality using individual-level data in a prospective cohort study which is population-based.

Generalized trust in other people was included as a covariate because it represents another dimension of trust than trust in the healthcare system. As stated in the introduction, generalized trust in other people constitutes horizontal trust, i.e. trust among equals without a power gradient, while trust in the healthcare system is an example of vertical trust, i.e. trust across a power gradient. Although generalized trust in others and trust in the healthcare system are significantly associated (see Table 1), the inclusion of generalized trust in other people in model 4 does not affect the results to any important extent because the two forms of trust represent two distinct and separate dimensions of trust. Age and sex were included as covariates as natural confounders. SES and country of birth were included as socioeconomic and additional demographic covariates. Chronic diseases was included as an indicator of health at baseline. Health-related behaviors were included as covariates because they may be both mediators and confounders of the associations.

Diagnoses were stratified broadly for the three cause-specific groups CVD, cancer and other causes mortality because they are the three main causes of death in present day Sweden and have been so for several decades. In fact, the broad distribution of causes of death in this study are very similar to the national distribution in official register data in Sweden. These findings suggest that this study is highly representative also regarding mortality patterns.

No significant results were observed for the no trust and do not know categories in the multiple adjusted models. The prevalence of these two categories are 3.2% and 0.9%, respectively. Furthermore, the no trust category is significantly and substantially younger than any of the other groups that are defined according to their trust in the healthcare system. Issues related to statistical power make it hard to interpret the findings for these two comparatively small groups. It is apparent that still almost 75% of the public aged 18–80 years have very high or rather high trust in the healthcare system, and that the major part of the remaining 25% have not particularly high trust.

Implications for policy and practice include the direct conclusion to reduce the queues in the healthcare system, particularly those involving clinical cancer investigations and treatments. The apparent queueing problems in the healthcare system have also triggered an ongoing debate regarding extensive structural reforms of the healthcare system. Some debaters, most notably the Christian Democratic Party among others, have advocated transferal of responsibility for the healthcare system from the 21 regions to the Swedish state in order to achieve higher efficiency and conformity nationally. This debate has been ongoing for a long time due to these apparent queueing problems.

Implications for research include the use of social and behavioral sciences in general and political science in particular to understand unexpected results in public health research. The logic of moderate or more limited trust following structurally adverse conditions in the healthcare system follows from the notion that such moderate/limited trust may be rational under certain practical circumstances and constant restrictions in access to healthcare. Following the same logic, Uslaner (2002) has suggested that moderate trust may be more rational in politics than unreservedly high trust, because moderate trust enhances critical thinking, questioning and fruitful debate. Still, it should be noted that complete absence of trust will have directly adverse effects and will importantly increase the risk of open antagonism and even violence. Moderate trust in combination with openness may under certain circumstances be optimal.

### 4.1 Strengths and limitations

The study is longitudinal, population-based and large with sufficient statistical power. The participation rate 54.1% is acceptable given similar participation in other public health questionnaire studies in the same timeframe (around 2008). The respondent population has also been judged as acceptably representative of the total population with regard to age, sex, education and country of birth, although with some underrepresentation of younger people, men, people with low education and born abroad. The risk of selection bias in the 2008 survey has been judged as comparatively small (Lindström & Rosvall, 2018).

As stated in the introduction, items on trust in healthcare and the healthcare system are considered valid indicators of medical performance (Chang et al., 2013; Straten et al., 2002). Socioeconomic status (SES) dimensions include occupation, income and education. These dimensions are considered mutually highly correlated although the correlation is not high enough for them to be considered identical. There were no questions concerning income in the public health questionnaire in Scania 2008. Education included as a covariate in the multiple survival regression models does not affect the results, but due to a higher number of internally missing it substantially increases the number of participants excluded from the analyses after restrictions. The LTPA item is acceptably valid and reliable with regard to golden standard items which measure four-day whole-day calorimetry, heart rate (monitoring), and double-labelled water (Wareham et al., 2003). The smoking item is valid and reliable (Wells et al., 1998). Swedish public register data are generally highly valid. The fact that the cardiovascular, cancer and other causes mortality categories of diagnoses are broad and mutually exclusive in combination with the high validity of public register data in Sweden means that the risk of misclassification is small.

Covariates and confounders including age, sex, SES, country of birth, chronic disease, LTPA, smoking, alcohol consumption and generalized trust in other people were adjusted for in the multiple analyses in Table 2, Table 3.

Proportionality has been extensively tested in this study, including analyses of all-cause mortality with all covariates in model 4 for 1.3-year, 2.3-year, 3.3-year and 4.3-year follow-up yielding similar strength and direction of effect measures (HRRs) across different follow-up periods. Proportionality was also tested with statistical tests and Schoenfeld's residuals, all tests leading to the same results, as expected.

## 5 Conclusion

The results of this study were unexpected because the groups with rather high and not particularly high trust in the healthcare system had significantly lower all-cause mortality than the reference group with very high trust in the healthcare system. These statistically significant results remained throughout the multiple analyses, and were explained by lower cancer mortality in both the rather high and not particularly high trust respondent groups, and also lower CVD mortality in the not particularly high trust respondent group. No significant results were observed in the adjusted models for other causes mortality. No significant results were observed for the no trust and don't know categories in the multiple adjusted models, but numbers in these groups were very small and thus hard to interpret. The results suggest a comparative advantage of moderate trust compared to very high trust in a setting characterized by restricted access to treatment particularly with regard to cancer and CVD treatment, but may not be generalizable to settings with no long-term queueing for cancer and CVD treatment.

## Ethical statement

The present study was approved by the Ethical Committee (*Etikprövningsnämnden*) in Lund (No. 2010/343).

## Funding

This study was funded by the 10.13039/501100004359Swedish Research Council (Vetenskapsrådet) (K2014-69X-22427-01-4), the 10.13039/501100004359Swedish Research Council (Vetenskapsrådet) (2019-01631) and the ALF government grants (ALF-medel).

## Declaration of competing interest

There are no conflicts of interest.
